# Nasal Muco-ciliary transport time alteration: efficacy of 18 B Glycyrrhetinic acid

**DOI:** 10.1186/s40248-017-0110-7

**Published:** 2017-11-29

**Authors:** Desiderio Passali, Chiara Cappello, Giulio Cesare Passali, Cemal Cingi, Codrut Sarafoleanu, Luisa Maria Bellussi

**Affiliations:** 1 ENT Clinic, Policlinico S. M. alle Scotte, Siena, Italy; 2 ENT Department, Sacred Heart University, Rome, Italy; 3 ENT Department, Faculty of Medicine, Eskisehir Osmangazi University, Eskisehir, Turkey; 4 ENT Dept, Santa Maria Hospital University of Medicine and Pharmacy, Bucharest, Romania

**Keywords:** MCT, Glycyrrhetinic acid, HMGB1, Sirt 6, Nasal inflammation

## Abstract

**Background:**

Mucociliary clearance is the main self-clearing system of the nasal cavity and paranasal sinuses.

This is a very important means of non specific defence against continuous organic and inorganic contamination conveyed by air. It works by trapping particles and microorganisms in the mucus and then by transporting the mucous film to the pharynx where it is eliminated with a cough or swallowed. Its congenital or acquired abnormalities are involved in the occurrence of widespread infectious and often severe nose and paranasal sinuses diseases; generally concerning the rhinopharyngealtubal district.

Restoring mucociliary clearance of the nasal epithelium when altered thus represents a key therapeutic tool against rhinosinus chronic diseases.

This study evaluates the clinical efficacy of the inhalation of a natural compound (Narivent® nasal spray) in chronic vasomotor rhinitis.

**Methods:**

The study involved 79 patients suffering from chronic vasomotor rhinitis presenting an increased mucociliary clearance time. Patients were randomized into 2 groups: a first group of 49 subjects and a second group of 30 subjects.

The first group was treated with a nasal spray (Narivent® nasal spray) (2 sprays per nostril twice a day) for 30 days.

The second group was treated with a nasal spray containing isotonic solution in the same way and for the same period of the first group.

Nasal Mucociliary transport time was measured in the patients of both groups before treatment, after 15 days of treatment, and at the end of the 30 days treatment.

**Results:**

The study shows how one of the treatments carried out determines a major objective reduction of the mucociliary clearance time in the patients under examination, using the method which involves the use of an insoluble coloured tracer (vegetable carbon), bringing the values back within normal range. At the end of the study we objectivated an increase in the rate of mucociliary transport in 97.9% percentage of patients we enrolled.

**Conclusions:**

This study shows the effectiveness of treatment with natural extracts with nasal mucosa restoring function in the treatment of chronic vasomotor rhinitis, a nasal inflammatory disease characterized by morphological and functional alteration of the normal nasal mucosa.

## Background

The mucosa of the nose and paranasal sinuses is formed by a respiratory cylindrical motile epithelium, completely analogous to the one of the trachea and bronchi.

Among the cells with cilia which, with their undulating movement, sweep nasal secretions from the outside towards the inside, there are numerous serummucous glands that produce nasal mucus, which has the function of retaining the dust particles inhaled and releasing moisture and heat to the breathed in air.

Nasal mucus is rich in glycoproteins and contains numerous molecules (IgA, lysozyme, defensins etc.) which contribute to a joint specific and nonspecific defence of the nasal epithelium. Its rheological properties influence the smooth operation of that function called mucociliary clearance.

This clearance particularly takes advantage of the mucociliary transport (MCT) system which allows all secretions to be pushed by the ciliated cells towards the back of the nasal passages to then descend into the pharynx.

The absence or deficiency of this self-clearing system of the nasal cavity and paranasal sinuses can constitute evidence or be the consequence of many diseases.

The present study was conducted to evaluate the clinical efficacy of nasal inhalation of a medical device (nasal spray) in selected patients with chronic vasomotor rhinitis, a nasal pathology which is characterised by an increased mucociliary transport time.

Narivent® is a natural compound with antioedema and anti-inflammatory osmotic properties mainly consisting of Mannitol, Glycerol and Dipotassium Glycyrrhizate.

The treatment effectiveness was evaluated following objective methods, with the measurement of nasal MCT speed before, during and after treatment.

During the recruitment and follow up visits, samples of nasal mucosa cells were taken by scraping, subsequently examined by optical microscope.

## Materials and methods

### Patients

The study involved 79 subjects suffering from chronic vasomotor rhinitis with increased mucociliary clearance time from a minimum of 24 min to over 30 min, between the ages of 16 and 61 years, of both genders. The subjects were randomised into two groups of 49 (first group) and 30 (second group) subjects.

The main recruitment criterion was the presence of chronic vasomotor rhinitis not associated with nasal polyposis but associated with a deficiency of mucociliary clearance. No other drugs were allowed during the treatment and in the 30 days before the beginning of the study.

The study was conducted by administering two sprays per nostril twice a day for 30 days the drug^1^ in the first group and an isotonic solution in the second group.

At the time of the recruitment visit, all personal data and clinical history were collected and ENT examination with a fiberoptic examination of the nasal cavity and mucociliary transport test (condensed results are reported in Table 1) were performed.

**Table 1:** Patients data

		Mean MCT	Responders	Normalization of MCT	σ	σ^2^
Group 1 (49 Patients)	Before treatment	28.7	**–**	**–**	1787	3194
	After 15 days of treatment	19.1	85.7% (42)	75.5% (37)	6542	42,802
	After 30 days of treatment	15.3	97.9% (48)	97.9% (48)	4234	17,934
Group 2 (30 Patients)	Before treatment	27.9	**–**	**–**	1905	3632
	After 15 days of treatment	26	56.66% (17)	13.3% (4)	4262	18,165
	After 30 days of treatment	24.3	83.33% (25)	30% (9)	4453	19,832

Patients of both groups were assessed before and after treatment by measuring MCT time.

A nasal scraping was performed before and after treatment on some consenting subjects (12 cases).

All patients recruited in the study were previously informed and gave their consent to participate in this study.

### Mucociliary clearance

Patients with rhinosinus pathologies often exhibit a decreased mucociliary clearance, evaluated as an increase in the mucociliary transport time, expressed in minutes.

The MCT time is calculated in our clinic by placing a tracking powder (vegetal charcoal powder) to the head of the inferior turbinate [1]; through direct pharyngoscopy it is then possible to detect the transit of the tracer from the posterior pharyngeal wall. The test requires quite some collaboration by the patient, who must keep a seated position without either blowing or sniffing. The charcoal powder used as a tracer, as it is insoluble and clearly visible in a pink environment such as that of the nasopharyngeal area, behaves as an inert material, adhering to the mucosa and transported passively by the movement of mucus and cilia. Our experience shows that this method for the measurement of MCT, compared to others at our provision such as isotopic measurement with iodine 131 / technitium 99, is reliable, low invasive and not expensive [1].

Normally, the tracer is found in the pharynx after about 10–15 min [2], but there are intersubjective and subjective variations, and only values higher than 25 min are considered pathological, and for values exceeding 30 min the mucociliary clearance is considered “blocked”.

The MCT time is calculated for each patient as the average of the values obtained in both nasal cavities.

### Analysis of the samples

Nasal scraping was carried out at the same time as the MCT test in 12 cases.

### Collection and analysis of cytology specimens

In this study, the collection of cytology specimens was performed noninvasively, by *nasal scraping.* After having the patient blown his/her nose in order to eliminate any excess secretions, the top layer of the mucous membrane at the level of the middle third of the inferior turbinate was removed bilaterally. An appropriate plastic *curette* was used. A sample was collected both from the right and from the left nostril for each patient.

The cellular material was then transferred and laid, swiping it as uniformly as possible, on a slide.

### Staining

We used the May Grunwald Giemsa rapid staining procedure (quick stain).

The test was carried out as follows: after drying the smear at room temperature, the slide is immersed 5 times for 1 s in the three solutions, one after the other, washing it under running water.

With this staining procedure the nuclei became purple, cytoplasmic filaments pink, eosinophil granules orange.

### Microscopic observation

Examination was carried out with a Nikon optical microscope at 100× magnification.

Each slide was evaluated dividing it into 6 equal fields in order to count the amount of each cell type per microscopic field. A comparison between the cells amount before and after treatment was then carried on.

### Statistical analysis

A clinical study in vivo was carried out making on a comparison between pre and after treatment phase with a mid therapy course assessment, based on objective clinical criteria.

The statistical significance of the results was assessed by a one way ANOVA analysis and two tailed T-Test.

## Results

The analysis of the results was conducted by analysing only those subjects who had completed the treatment and had carried out the recruitment and control visits.

The condensed data collected in this study are listed in Table 1.

The analysis of the results is shown in Table 2.

**Table 2:** Results: ANOVA test in both groups and final comparative T-Test

First group ANOVA test	Second group ANOVA test
f-ratio = 108,02377	f-ratio = 6,78,346
*p* <0,00001	*p* = 0.001829 (<0.05)
Comparison between groups	
t = 8,91,363	
* p* <0.00001	

In both groups of patients included in this study the average mucociliary transport time (MCT) detected during/before treatment was around 28 min. (28.8 min. in the first group and 28 min. in the second group) with a minimum value of 24 min.

31 out of the 49 cases included in the first group of study (63.3%) and 11 out of the 30 subjects included in the second group (36.7%) had a blocked MCT (> 30 min.).

At the end of the treatment no adverse reactions were reported.

In the first group the results were the following.

A MCT mean time of 19.2 min. with a minimum value of 8 min. was found at the first follow up exam, performed after 15 days of continuous therapy, thus a mean decrease of 9.6 min. representing a 33.3% decrease from the starting value. The percentage of responder patients was equal to 85.7%. In 75.5% of the examined cases we witnessed a normalisation of the MCT time. In 5 cases, MCT time remained blocked.

The results of the final follow up exam, carried out after 30 days, at the end of therapy, show an average MCT time of 15.3 min. with a minimum value of 10 min., a final average decrease of 13.4 min. representing a 46.7% decrease from the starting value and of 20% compared with the previous follow up. The percentage of patients where there was an increase in the rate of mucociliary clearance was 97.9%. Only in one case we observed persisted in having a MCT time > 30 min.

Therefore, the treatment was successful in 97.9% of cases, bringing the MCT time within the threshold of normality in 48 out of 49 cases. Almost all patients answering to a simple questionnaire referred a favourable opinion on the treatment and reported an improvement in their nasal symptoms and especially on nasal obstruction almost accordingly with the MCT time improvement.

At the end of the observation period there was only one non responder patient documented.

The data analysis performed using the ANOVA method was statistically significant (*p* <0.00001).

In the second group the results were the following.

A MCT mean time of 26 min. with a minimum value of 10 min. was found at the first follow up exam, performed after 15 days of continuous therapy, thus a mean decrease of 1.9 min. representing a 6.9% decrease from the starting value. The percentage of responder patients was equal to 56.6%. In 13.3% of the examined cases we witnessed a normalisation of the MCT time. Only in one case MCT time remained blocked.

The results of the final follow up exam, carried out after 30 days, at the end of therapy, show an average MCT time of 24.4 min. with a minimum value of 10 min., a final average decrease of 3.6 min. representing a 12.9% decrease from the starting value and of 13.8% compared with the previous follow up. The percentage of patients where there was an increase in the rate of mucociliary clearance was 83.3%.

The treatment was completely successful in 30% of cases, bringing the MCT time within the threshold of normality in 9 out of 30 cases.

At the end of the observation period there were four non responder patients documented.

The data analysis performed using the ANOVA method was statistically significant (*p* <0.05).

## Discussion

Some studies report how the effects of the most common formulations of nasal sprays and solutions used in clinical practice for treating patients suffering from nose/sinus disorders (isotonic or hypertonic saline solution, Ringer’s lactate and cortisone formulations) result in an improvement in the symptoms and quality of life, but not in a significant decrease in mucociliary transport time [3, 4].

A 2008 systematic review published in the Cochrane Library analysed more than 2000 articles, including 64 clinical trials, on the efficacy of nasal washes to treat the symptoms of chronic rhinosinusitis. The conclusions highlighted that the use of hypertonic saline solution promotes mucociliary clearance, but there is no evidence of a greater effectiveness compared with the isotonic solution or with other agents in relieving rhinosinusitis symptoms [5].

In this regard, a trial on human nasal mucosa models carried out by Kim and colleagues shows that given the lack of a direct correlation between ciliary movement frequency in vitro and mucociliary clearance in vivo and the presence of contradictory results in the literature on the effects of irrigation with various concentrations of saline solutions on ciliary activity, the most important factors which determine mucociliary clearance are the quantity and the rheological properties of the secreted mucus [6].

This study also states that "the isotonic saline solution is the most physiologic irrigation solution in terms of cell morphology and mucin secretion from airway epithelial cells". Washes of distilled water or hypotonic solutions, in fact, harm the integrity of the cell junctions creating disruptions in the mucosal barrier, promoting the secretion of large amounts of mucus and decreasing the total number of ciliated cells, while the action in vitro of hypertonic saline solutions promotes the secretion of mucins, albeit without damaging the nasal mucosa significantly from a morphological point of view.

**Fig. 1:**
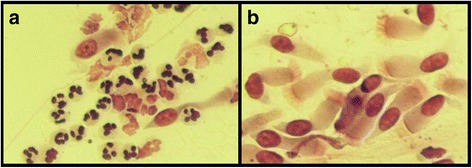
**a** Neutrophil rhinopathy before treatment. **b** After treatment normalization of rhinocytology: ciliate cells of nasal epithelium

**Fig. 2:**
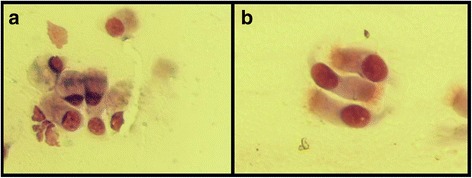
**a** Before treatment muciparous rhinopathy. **b** After treatment: restoration of physiological rhinocytology with normal ciliate cells

The optical microscope analysis of the nasal mucosa samples obtained from 12 subject by scraping highlights the normalization of the after treatment nasal cytology (Fig. 1a-1b and 2a-2b).

The therapy cycle was well tolerated, with no side effects reported by patients.

This study allows underlining how the administration of the medical device under consideration improves an important nasal functionality parameter, such as mucociliary clearance is and should be regarded. In addition, it shows that this improvement is statistically significant in comparison with the improvement achieved in the second group (*p* <0.05).

The reason for this beneficial effect is justified by our present knowledge on the importance of HMGB1 (High Mobility Group Box 1) during an inflammatory process and on the inhibiting action of glycyrrhetinic acid on it [7–14].

HMGB1 is a non histonic nuclear protein that is primarly expressed in the nucleus of normal nasal mucosa cells. Higher extracellular levels and lower nuclear levels of HMGB1 are normally found in chronic inflammatory conditions [15].

It has been recently observed that HMGB1 extracellular release is related to the nuclear concentration of Sirt 6, a protein member of Sirtuines family, NAD+ dependent class III histone deacetylases closely correlated to inflammatory conditions [15–17].

Sirt 6 is found in the nucleus of normal nasal mucosa cells. It promotes epithelialization of nasal mucosa. A depletion of this protein induces a dramatic translocation of HMGB1 from nucleus to cytoplasm and leads to the suppression of the number of human nasal epithelial cell cilia [15]. It has recently been observed [15] that glycyrrhetinic acid increases also Sirt6 protein levels and activity.

## Conclusions

This study shows the effectiveness of a natural extract in the treatment of chronic vasomotor rhinitis. Glycyrrhetinic acid is a specific medical compound that may be isolated from the licorice plant that binds selectively to HMGB1 protein released extracellularly and so inhibits its cytokine activities through a scavenger mechanism on the protein accumulation [7, 15]. Glycyrrhetinic acid increases also Sirt6 protein levels and activity, decreasing HMGB1 release and promoting nasal epithelial cells differentiation stimulating ri-epithelialization of nasal mucosa and increasing the number of cells cilia.

## Abbreviations

HMGB1: High Mobility Group Box 1
MCT: Muco-Ciliary Transport

## Footnotes

1 Narivent® nasal spray
